# A study on relationship between elderly sarcopenia and inflammatory factors IL-6 and TNF-α

**DOI:** 10.1186/s40001-017-0266-9

**Published:** 2017-07-12

**Authors:** Ai-Lin Bian, Hui-Ying Hu, Yu-Dong Rong, Jian Wang, Jun-Xiong Wang, Xin-Zi Zhou

**Affiliations:** Department of Geratology, Tianjin First Central Hospital, No. 24 of Fukang Road, Nankai District, Tianjin, 300192 China

**Keywords:** Elders, Skeletal muscle reduction, IL-6, TNF-α

## Abstract

**Background:**

This report aims to study the relationship between sarcopenia of elderly in community and inflammatory factors IL-6 and TNF-α.

**Methods:**

A total of 441 elders who undertook physical examinations were included into this study. The age of these subjects were >60, in which 235 subjects were male and 206 subjects were female. According to the diagnostic standards of sarcopenia set by EWGSOP and AWGS, these subjects were divided into two groups: sarcopenia, and non-sarcopenia groups. The living habits, disease status, biochemical indexes, and levels of IL-6 and TNF-α of these subjects were investigated.

**Results:**

The morbidity rate of sarcopenia was 17.02% in male subjects and 18.9% in female subjects. In elderly subjects >80 years old, morbidity rate was 25.3% in male subjects and 35.1% in female subjects. The history of smoking in patients with sarcopenia was long, and their regular exercise history was short (*P* < 0.01). Furthermore, differences in handgrip strength (HG), fat-free mass (FFM), bone mineral content (BMC), plasma albumin (ALB) and serum creatinine (Cr), and body fat content (FAT) in patients between the sarcopenia and non-sarcopenia groups were statistically significant (*P* < 0.05). Moreover, differences in IL-6 and TNF-α levels between these two groups were statistically significant (*P* < 0.05). In addition, BMI was positively correlated to TNF-α levels, and ALB was negatively correlated to IL-6; while BMI and VFA were positively correlated to TNF-α levels, and SMM, HDL-C, Hb, HG were negatively correlated to IL-6 level (*P* < 0.05). Multiple linear regression analysis suggested plasma ALB and BMI were the independent risk factors of TNF-α, while VFA was the independent risk factor of IL-6.

**Conclusions:**

The onset of sarcopenia was associated with poor exercise habits, disease history, and nutritional status. The emergence of sarcopenia was accompanied by increased levels of inflammation factors TNF-α and IL-6. Plasma albumin, BMI, and VFA were inflammatory factor predictors of TNF and IL-6.

## Background

With the rapid aging of the population, chronic diseases in the elderly have become a major social problem worldwide. Among the elderly diseases, in addition to diabetes, cardio-cerebrovascular diseases, tumors and other frequently occurring diseases, the health problems caused by changes in the body compositions of the elderly are easy to be neglected. As the body aged, the composition of the body also changed such as the increase in adipose tissues and reduction in muscle tissues. Foreign studies have shown that age-induced sarcopenia has become an important health problem that affects the quality of life of the elderly. In 1989, Ronsenberg first proposed the definition of sarcopenia, that is, a progressive decline in skeletal muscle mass and strength. Its negative effects on the elderly could be observed in many aspects: increased risk of falling down and fracture, increased re-admission rates, and increased mortality [1]. Studies have revealed that age-related body composition changes, as well as chronic inflammation and proinflammatory factors produced by adipose tissues, are closely related to sarcopenia [2–4]; and this inflammatory reaction may play a key role in the occurrence and development processes of sarcopenia. The purpose of this study is to investigate the relationship between changes in body composition and the incidence of sarcopenia vs. inflammatory factors interleukin-6 (IL-6) and tumor necrosis factor-α (TNF-α).

## Methods

### Research subjects

This cross-sectional study selected a total of 441 elderly subjects >60 years old, who underwent physical examinations in Tianjin First Central Hospital between May 2015 and September 2015. Among these subjects, 235 subjects were male (mean age: 75.31 ± 8.29 years old) and 206 subjects were female (mean age 75.30 ± 3.46 years old). Furthermore, among these subjects, 161 subjects were ≥80 years old, which included 87 male subjects and 74 female subjects.

### Inclusion criteria

Subjects >60 years old, who could walk by themselves or stand for 5 min with other auxiliaries, and had no history of mental illness were enrolled into this study.

### Exclusion criteria

(1) Subjects with tumor or severe weakness. (2) Subjects with diseases that seriously affect their health such as serious arrhythmia, heart failure, infection, edema, or massive ascites. (3) Subjects with endocrine or metabolic diseases that failed to be well-controlled such as type-2 diabetes and thyroid gland disease. (4) Subjects who were taking anti-inflammation drugs. (5) Autoimmune diseases.

### Diagnostic criteria and grouping of sarcopenia subjects

#### Diagnostic criteria

The diagnostic criteria were based on the diagnostic criteria of sarcopenia established by the European Working Group on Sarcopenia in Older People (EWGSOP) in 2010 and the Asian Working Group for Sarcopenia (AWGS) in 2015, which include the decrease in muscle mass, muscle strength, and/or body function. Muscle mass was evaluated using the appendicular skeletal muscle index (ASMI), which is equal to the appendicular skeletal muscle mass (ASM)/the square of the height (m^2^). The standard evaluation is that the value is less than two mean standard deviations (SD) of the mean value of the youth with the same race and gender, combined with the recommendations of AWGS and EWGSOP. A value <7.0 kg/m^2^ for male subjects and a value <5.7 kg/m^2^ in female subjects were adopted as ASMI cut-off values, respectively. Handgrip strength (HG) is an indicator of muscle strength. A HG strength value <26 kg for male subjects and a HG strength value <18 kg for female subjects were set as cut-off values.

#### Grouping method

All subjects were divided into two groups, according to ASMI and HG strength:

1. Sarcopenia group: In male subjects, ASMI was <7.0 kg/m^2^ and HG strength was <26 kg. In female subjects, ASMI was <5.7 kg/m^2^ and HG strength was <18 kg.
2. Non-sarcopenia group: In male subjects, ASMI was ≥7.0 kg/m^2^, and in female subjects, ASMI was ≥5.7 kg/m^2^.

### Detection index

#### Body composition analysis

Bioelectrical impedance analysis (BIA) was employed to measure the composition of the human body using an InBody S10 body composition analyzer (Biospace Co., Ltd., South Korea). Measurement indexes included the following: intracellular water (ICW), extracellular water (ECW), appendicular skeletal lean mass (SLM), body fat content (FAT), fat-free body mass (FFM), bony mineral content (BMC), protein content (pro), body cell mass (BCM), body mass index (BMI), arm circumference (AC), arm muscle circumference (AMC), and waist circumference (WC).

#### Body measurements

1. Body weight (kg), height (CM), and blood pressure (mmHg) measurements;
2. HG strength determination: HG strength was determined using a hand-muscle developer (WCS-II, Beijing). Each hand was tested twice, the maximum value was used, and these values were corrected to 0.1 kg.

#### Determination of laboratory indicators

##### Clinical inspection indexes

Subjects fasted for 8 h. In the morning, 5 ml of fasted elbow venous blood was drawn into a vacuum mining vessel, placed in static for 30 min, and the serum was isolated at 4 °C. Indicators included routine blood, including hemoglobin (Hb), and lymphocyte (Lym) and blood biochemistry, including total cholesterol (TC), triacylglycerol (TG), blood glucose (Glu), serum albumin (ALB), and creatinine (Cr). The detection of the above indicators was completed on the same day. At the same time, another 400 μl of serum was packed into two Eppendorf tubes, placed in the refrigerator at −70 °C, and were prepared for the batch determination of IL-6 and TNF-α.

##### Detection of inflammatory factors

1. Detection of TNF-α: TNF-α was determined by solid-phase sandwich enzyme-linked immunosorbent assay. The kit was produced by RapidBio (RB, USA).
2. Detection of IL-6: IL-6 was determined by the competitive inhibition of enzyme-linked immunosorbent assay. The kit was produced by RapidBio (RB, USA).

### Social demographic factors and way of life

Social demographic factors were obtained through questionnaires, which included marital status, living environment, smoking history, drinking history, and daily exercise. Previous medical history included hypertension, coronary heart disease, diabetes, and cerebral infarction history.

1. Definition standards of regular exercise: Exercise >3 times a week for >30 min each time, which persisted for at least 6 months. All these above were considered as regular exercise.
2. Definition standard of smoking: According to the definition of active smokers and passive smokers established by the World Health Organization (WHO), that is, people who have smoked for six consecutive months or six cumulative months, or smoked more than 6 months in a lifetime were defined as cigarette smokers. These subjects were divided into two categories: smokers and non-smokers.
3. Definition standard of drinking: These subjects were divided into two categories, according to alcohol drinking habits: drinkers and non-drinkers.

### Statistical methods

Data were analyzed using Windows SPSS 19.0 statistical software package. Normal distributed data were expressed as mean ± standard deviation (*x* ± SD), and were analyzed by *t* test. Count data were analyzed by *X* ^*2*^-test. Correlations were analyzed by Spearman’s rank correlation method. Linear regression and multiple linear regression equations were used for multivariate analysis. *P* < 0.05 was considered statistically significant.

## Results

1. Among these 441 subjects, 79 subjects had sarcopenia including 40 male subjects (17.02%) and 39 female subjects (18.9%). Furthermore, among these 79 subjects, 48 subjects were >80 years old including 22 male subjects (25.3%) and 26 female subjects (35.1%). Comparisons on the general information, life habits, disease history, body composition, and biochemical indicators of patients between these two groups are listed in Table 1. In life habits, patients with sarcopenia had a long history of smoking and less regular exercise, compared with non-sarcopenia patients; and there was a significant difference between these two groups (*P* < 0.01). For status of illness, more patients suffered from coronary heart disease in the sarcopenia group, and the difference was statistically significant between these two groups (*P* < 0.05). For body composition, differences in height, weight, HG strength, ICW, ECW, pro, FFM, and BMC in male subjects and height, HG strength, ICW, ECW, pro, FAT, FFM, BMC, and VFA in female subjects between the sarcopenia and control groups were statistically significant (*P* < 0.01). For clinical biochemical indexes, differences in levels of DBP, ALB, and Cr in male subjects and levels of DBP, ALB, Cr, and Hb in female subjects between the sarcopenia and control groups were statistically significant (*P* < 0.05).

**Table 1:** A comparison of clinical data between muscle decrease disease group and non-muscle decrease disease group

Observation index	Muscle decrease disease (*n* = 79)		Muscle decrease disease (*n* = 362)	
	Man (*n* = 40)	Female (*n* = 39)	Man (*n* = 195)	Female (*n* = 167)
ASMI	7.69 ± 1.02	5.27 ± 0.81	8.25 ± 1.34	7.07 ± 1.27
Age	70.88 ± 7.23	79.78 ± 4.32	73.54 ± 7.81	80.82 ± 8.34
Height	166.28 ± 5.31*	153.72 ± 5.30*	171.82 ± 8.10	160.95 ± 5.84
Weight	67.43 ± 10.73*	59.34 ± 17.11	73.35 ± 11.34	61.90 ± 9.15
HG	30.91 ± 7.62*	19.65 ± 6.96*	33.8 2 ± 8.14	21.17 ± 5.52
ICW	23.18 ± 4.03*	15.67 ± 2.64*	25.89 ± 4.50	19.46 ± 3.00
ECW	14.58 ± 2.34*	10.58 ± 1.69*	16.51 ± 2.38	12.36 ± 1.55
Pro	10.01 ± 1.74*	6.77 ± 1.14*	11.19 ± 1.95	8.41 ± 1.29
FAT	16.33 ± 8.91	23.76 ± 13.32*	16.26 ± 7.76	18.70 ± 7.78
FFM	51.10 ± 8.19*	35.59 ± 5.63*	57.10 ± 9.49	43.20 ± 6.19
BMI	24.38 ± 3.59	24.88 ± 5.71	24.98 ± 5.20	23.92 ± 3.52
BMC	2.66 ± 0.41*	2.09 ± 0.24*	3.04 ± 0.58	2.42 ± 0.32
AC	33.01 ± 9.83	29.76 ± 4.61	32.85 ± 7.00	30.17 ± 2.92
AMC	27.99 ± 9.75	23.06 ± 2.67	27.82 ± 5.44	24.09 ± 1.97
WC	83.34 ± 10.90	90.44 ± 21.30	86.33 ± 10.55	84.37 ± 9.70
VFA	75.74 ± 47.44	142.21 ± 82.66*	75.54 ± 38.10	95.36 ± 50.33
SBP	136.28 ± 18.71	137.00 ± 21.4	136.04 ± 21.62	132.66 ± 38.17
DBP	79.40 ± 10.32*	75.00 ± 7.50*	78.28 ± 12.42	74.45 ± 21.24
ALB	39.95 ± 5.81*	35.51 ± 7.55*	41.59 ± 4.02	41.74 ± 3.79
Cr	94.15 ± 27.99*	45.82 ± 29.07*	89.40 ± 24.05	24.05 ± 43.53
Glu	5.67 ± 1.43	5.04 ± 1.17	5.71 ± 1.92	5.68 ± 2.21
TC	4.45 ± 1.15	4.02 ± 1.19	4.40 ± 1.21	1.1 ± 0.19
TG	1.14 ± 0.51	1.13 ± 0.51	1.32 ± 0.67	1.72 ± 1.22
Lym	1.79 ± 0.68	1.62 ± 0.59	2.04 ± 0.69	1.88 ± 0.65
Hb	135.83 ± 22.02	118.33 ± 26.37*	140.06 ± 16.16	131.74 ± 13.57
Hyper (%)	15 (37.5.0)	15 (38.4)	76 (39.0)	70 (42.1)
Cardiac (%)	19 (48.0)*	25 (66.7)*	47 (24.3)	43 (26.3)
NC (%)	5 (12.5)	4 (10.2)	33 (17.0)	16 (10.5)
DM (%)	10 (25.0)	9 (23.1)	41 (21.1)	35 (21.0)
Smoke (%)	5 (12.0)*	2 (4.0)*	19 (9.7)	0 (0)
Drink (%)	11 (28.0)	0 (0)	48 (25.2)	0 (0)
Sport (%)	4 (10.0)*	5 (12.8)*	34 (17.4)	58 (34.7)

* *P* < 0.05, there were significant differences between the groups
  *Height, the height of a person; weight, body weight; HG, handgrip or handgrip strength; ICW, intracellular water; ECW, extracellular water; pro, protein; FAT, fat content of the body; FFM, fat-free body weight; BMI, body mass index; BMC, bone mineral content; AC, upper arm circumference; AMC, arm muscle circumference; WC, waist circumference; VFA, visceral fat area; SBP, systolic blood pressure; DBP, diastolic blood pressure; ALB, plasma albumin; Cr, serum creatinine; Glu, blood glucose; TC, total cholesterol; TG, triglyceride; Lym, blood lymphocyte count; HB, hemoglobin; Hyper, hypertension; Cardiac, coronary heart disease; NC, cerebral vascular disease; DM, diabetes mellitus; Smoke, smoking history; Drink, drinking history; Sport, exercise history.
2. Correlation analysis of body composition and sarcopenia is shown in Table 2. ICW, ECW, Pro, FFM, BMC, AC, and AMC body compositions were correlated to sarcopenia, and these correlations were significantly positive (*P* < 0.01). However, FAT had a significant negative correlation with sarcopenia (*P* < 0.05).

**Table 2:** Correlation analysis of Spearman with body composition and muscle decrease

	r	*P*
ICW	0.760	0.000*
ECW	0.811	0.000*
Pro	0.762	0.000*
FAT	−0.307	0.040*
FFM	0.780	0.000*
BMI	0.306	0.078
BMC	0.674	0.000*
AC	0.483	0.004*
AMC	0.578	0.000*
WC	0.160	0.366
VFA	−0.242	0.167

* *P* < 0.05, there was a significant difference
3. Correlation analysis of sarcopenia and blood biochemical indexes is shown in Table 3. There was a significant positive correlation between sarcopenia and the indexes of DBP, ALB, and Cr (*P* > 0.05).

**Table 3:** Correlation analysis between Spearman and biochemical indexes of muscular disorders

	r	*P*
SBP	0.071	0.692
DBP	0.343	0.047*
ALB	0.367	0.035*
Cr	0.420	0.015*
Glu	0.202	0.259
TC	0.223	0.211
TG	0.162	0.367
Lym	0.291	0.101
Hb	0.274	0.123

* *P* < 0.05, there was a significant difference
4. Correlation analysis of inflammatory factor and other indicators: The levels of inflammatory factors IL-6 and TNF-α in the sarcopenia and control groups are shown in Table 4. IL-6 and TNF-α levels in the Sarcopenia group were higher than the control group; the comparison between groups showed significant difference (*P* < 0.05). Correlation analysis of TNF-α, IL-6 levels and body composition, biochemical index is shown in Table 5. BMI was significant positively correlated with TNF-α, and ALB was significant negatively correlated with TNF-α (*P* < 0.05). BMI and VFA were significant positively correlated with IL-6, whereas SMM, Hb, HDL-C, and HG were significant negatively correlated with IL-6 (*P* < 0.05).

**Table 4:** Comparison of levels of inflammatory cytokines

Inflammatory factors	Muscle decrease disease group	Non-muscle decrease disease group	*P*
TNF-α (pg/ml)	165.39 ± 19.49	148.79 ± 26.06	0.01
IL-6 (pg/ml)	49.77 ± 22.14	39.72 ± 29.53	0.03

**Table 5:** Relationship between inflammatory factors and body composition and biochemical indexes

Variable	TNF-α		IL-6	
	r	*P*	r	*P*
ALB	−0.266	0.024*	−0.044	0.373
SMM	0.184	0.087	−0.238	0.039*
HDL-C	−0.099	0.233	−0.389	0.002**
Hb	0.034	0.400	−0.304	0.011*
BMI	0.346	0.008**	0.373	0.004**
VFA	0.212	0.059	0.464	0.000**
LDL-C	0.162	0.117	0.020	0.440
HG	0.411	0.070	−0.450	0.048*

* *P* < 0.05, there was a significant difference
5. Multivariate linear regression analysis: With TNF-α and IL-6, respectively, as the dependent variables, with BMI, SMM, VFA, ALB, HDL-C, LDL-C, and Hb as independent variables in the Logistic multivariate regression model, the results are shown in Table 6. It suggested that ALB and BMI had effective predictive value for TNF-α (*P* < 0.05), and VFA was the independent risk factor for the development of IL-6 (*P* < 0.05).

**Table 6:** Logistic regression analysis of inflammatory cytokines and other indexes

	TNF-α			*P*	IL-6			*P*
	*β*	Std	*t*		*β*	Std	*t*	
Constant	−268.119	145.975	−1.837	0.072	−9.738	93.284	−2.347	0.021
ALB	−7.763	3.045	−2.550	0.014*	1.638	1.946	0.842	0.404
BMI	9.192	3.036	3.027	0.004*	1.183	1.940	0.609	0.545
SMM	−0.522	1.255	−0.416	0.679	−0.325	0.802	−0.405	0.687
LDL	5.160	7.027	0.734	0.466	0.280	4.491	0.062	0.951
HDL-C	14.285	21.176	0.675	0.503	17.458	13.532	1.290	0.203
Hb	−0.846	0.511	−1.655	0.105	−0.129	0.327	−0.395	0.695
VFA	0.357	0.516	0.692	0.492	0.644	0.187	3.445	0.001*

* *P* < 0.05, there was a significant difference

## Discussion

Muscle reduction syndrome was defined as sarcopenia for the first time by Rosenberg in 1989 in the consensus published by EWGSOP in 2010 and AWGS in 2015. Since then, more attention has been given to sarcopenia by global medical communities; and strategies for diagnosis and treatment have been gradually improved. Sarcopenia is a prevalent disease in the elderly. The results of latest studies have revealed that the morbidity rate of sarcopenia was 5–13% in people aged between 60 and 70 years, and that this rate was 11–50% in populations of over 80 years [5, 6]. Since this can increase falling down and fracture risks in the elderly, and is closely related to the increase in disability and mortality rates [7–9], it is very necessary to explore the characteristics and pathogenesis of this disease to reduce its occurrence and development, so as to improve the life quality of the elderly and reduce the re-admission rate and medical cost. The reason why the elderly are prone to suffer from sarcopenia may be related to multiple factors [1, 10], such as abnormal protein metabolism, oxidative stress, and chronic inflammations. Based on the findings of this study, specific analyses are as follows.

### Risk factors of sarcopenia

Studies have shown that smoking and poor exercise habits were related to sarcopenia, which was consistent with previous research results. A large-scale study on sarcopenia in the elderly in Hong Kong indicated that smoking is one of the risk factors of sarcopenia. It was also found that quitting smoking could increase the amount of muscle content, fat content, and weight. Therefore, smoking cessation may be one of the means to prevent and treat sarcopenia. Exercise is also an effective means to prevent and treat sarcopenia, which can change the quality and strength of skeletal muscles, improve balance ability, and play a good role in the prevention and treatment of sarcopenia in the elderly. Furthermore, resistive exercise can increase the cross-sectional area and number of muscles through the reduction of body fat, especially subcutaneous and abdominal fats [11], which result in increased muscle mass. Li et al. found that resistance training can effectively increase the muscle content of limbs and reduce the level of inflammatory factors [12]. The serum level of IL-6 may be decreased by 14.4% in an elderly who can conduct regular exercises for 24 weeks. Therefore, exercise can reduce the level of serum IL-6 and inflammatory reaction to reduce the incidence of sarcopenia.

### Relationship between inflammatory factors and sarcopenia

Our research results indicated that IL-6 and TNF-α serum levels in elderly subjects with sarcopenia were higher than levels in the control group, and BMI VFA were independent risk factors for inflammatory cytokines IL-6 by multiple regression analysis. These were consistent with the results of a previous similar study [13]. As age increased, a chronic low-level systemic inflammatory reaction occurred in body; and inflammation is a concomitant reaction of cell senescence and organism aging. It was found in the study that, accompanied with organism aging, inflammatory factors in vivo increased significantly, and serum proinflammatory factors such as TNF-α, IL-6, and C-reactive protein (CRP) could increase 2–4 times, even in elderly without chronic diseases. Visceral fat tissue (VFA) is known to be distributed predominantly in the abdominal cavity. Recently, evidence supports that visceral fat tissue contains large number of adipocytes and is more hormonally active since there are more glucocorticoid and androgen receptors in comparison with subcutaneous fat [14]. Another evidence suggests that as adipose tissue increases the amount of the anti-inflammatory cytokine decreases, whereas there is an increase in the level of the proinflammatory molecules such as leptin, TNF-α, IL-1, and IL-6 [15]. In the elderly, increased serum levels of inflammatory factors were closely related to the reduction of skeletal muscle content and strength induced by senescence [16, 17]. Moreover, increased serum IL-6 levels are positively associated with disability rate and mortality [18], and negatively correlated to the recoveries of walking and muscle function in the fracture healing process in elderly women [19]. These large-scale study results revealed that supersensitive CRP and IL-6 are important predictors of muscle strength reduction within the next 3 years of middle- and old-aged people over 55 years [20]. Therefore, inflammatory reaction plays an important role in the development of elderly sarcopenia.

At the same time, our study found that ALB was an effective predictor of inflammatory factor TNF-α. Sullivan’s study found that inflammation appears to be a more powerful determinant of albumin, particularly CRP, and IL-6, and its change during the hospitalization than is nutrient intake in elderly patients admitted to a TCU [21]. Another study found, TNF-α, IL-10, and markers of oxidative stress were in negative correlation with HDL cholesterol and albumin. Resolution of the inflammation is probably the primary prerequisite to the normalization of the serum albumin [22].

According to the findings in the study, the possible mechanisms of IL-6 in the induction of sarcopenia are as follows. (1) Inflammatory factors can inhibit the synthesis of muscle proteins, accelerate protein decomposition, and upregulate the expression of muscle growth inhibitory factor myostatin and muscle atrophy proteins, F-box-1 Atrogin-1, etc., so as to accelerate protein catabolism and promote skeletal muscle consumption [23, 24]. Muscle tissues contain the most amounts of proteins in the human body, and IL-6 can lead to the reduction of muscle mass by means of disrupting protein synthesis and directly participating in protein decomposition. Furthermore, high levels of IL-6 can inhibit the anabolism of muscle tissue in IGF-I [25, 26]. (2) Age-related changes in body composition can easily cause increased cytokine products. As an organism ages, adipose cells increases, and IL-6 and TNF-α can easily be secreted, which aggravate inflammatory reactions. Furthermore, this would lead to the reduction in muscle mass and strength, resulting in the occurrence of sarcopenia [1, 10]. (3) Insulin resistance is another mechanism of sarcopenia. Insulin not only has the function of reducing blood sugar, but also accelerates the protein synthesis of targets on which the muscle fibers act. At the same time, the uptake of calcium in cells is affected by insulin. Insulin resistance may lead to decreased calcium uptake, which is not conducive to muscle contraction [27]. Therefore, elevated IL-6 and TNF-α serum levels can lead to insulin resistance and the occurrence of sarcopenia. (4) Recent studies have indicated that inflammatory reactions may also lead to the occurrence of sarcopenia through triggering mitochondrial abnormalities in the manner of disturbing the number or origin of mitochondria [28]. A study on the correlation between TNF-α and sarcopenia revealed that elevated levels of TNF-α are associated with the decline in muscle mass and strength. In addition, a study on 5-year continuous observations on elderly subjects aged within 70–79 years and 4-year continuous observations on elderly subjects aged >85 years has indicated that plasma TNF-α levels can predict the decline in muscle strength; wherein, as every standard deviation of TNF-α level increased, 1.2–1.3 kg of HG strength would be decreased [29].

Inflammatory reaction is just one of the factors for the occurrence of sarcopenia. Other factors include nutrition issues and hormone level decline. Sarcopenia would become one of the main reasons of disability and reduction in the quality of life in the elderly. In recent years, although many scholars have explored this in various aspects, its pathological mechanism remains unclear and there are no standard diagnostic criteria. In order to determine the effective method for preventing and treating this disease, it is necessary to proceed with more large-scale clinical studies.

## Conclusions

The occurrence of elderly sarcopenia is related to poor exercise habits, disease history, and nutritional status. The emergence of Sarcopenia was accompanied by increased levels of inflammation factors TNF-α and IL-6. Plasma albumin, BMI, and VFA were inflammatory factor predictors of TNF and IL-6. Prohibiting smoking habits and exercise could reduce the incidence of sarcopenia.

## Abbreviations

HG: handgrip strength
FFM: fat-free mass
BMC: bone mineral content
ALB: plasma albumin
CrL: serum creatinine
FAT: body fat content
IL-6: inflammatory factors interleukin-6
TNF-α: tumor necrosis factor-α
ASMI: appendicular skeletal muscle index
SD: standard deviations
BIA: bioelectrical impedance analysis
ICW: intracellular water
ECW: extracellular water
SLM: appendicular skeletal lean mass
SMM: skeletal muscle mass
FAT: fat content
FFM: fat-free body mass
BMC: bony mineral content
Pro: protein content
BCM: body cell mass
BMI: body mass index
AC: arm circumference
AMC: arm muscle circumference
WC: waist circumference
Hb: hemoglobin
Lym: lymphocyte
TC: total cholesterol
TG: triacylglycerol
Glu: glucose
ALB: serum albumin
Cr: creatinine
